# FRAILTY AND PRECARITY: UNTANGLING THE CONCEPTUAL PATHWAYS OF NEED AND DISADVANTAGE IN LATE LIFE

**DOI:** 10.1093/geroni/igz038.2185

**Published:** 2019-11-08

**Authors:** Amanda M Grenier

**Affiliations:** McMaster University, Health Aging and Society, Ontario, Canada, Canada

## Abstract

The concepts of frailty and precarity circulate in social gerontology and studies of aging, with the former a dominant construct, and the latter emerging as a way of linking experiences, insecurities and risks. Although these concepts are used inter-changeably by some authors, their roots, key areas of focus and meanings differ. This paper considers the state of knowledge on frailty, and sets this against the uses of precarity. A After outlining a recent scoping review on precarity that revealed a high number of articles cross-referencing concepts of frailty and vulnerability. the paper distinguishes key aspects of frailty, vulnerability, and precarity. Situating qualitative experiences of each serves as a means to further explore similarities and differences. The paper concludes with reflections on what (if anything) each of these allied concepts may offer understandings of late life, and in particular, the study of disadvantage across the life course and into late life.

